# HIV pretreatment drug resistance among cisgender MSM and transgender women from Lima, Peru

**DOI:** 10.1002/jia2.25411

**Published:** 2019-11-27

**Authors:** William L Trebelcock, Javier R Lama, Ann Duerr, Hugo Sanchez, Robinson Cabello, Trupti Gilada, Patricia Segura, Sari L Reisner, Kenneth H Mayer, James Mullins, Rachel A Bender Ignacio

**Affiliations:** ^1^ University of Washington Seattle WA USA; ^2^ Asociación Civil Impacta Salud y Educación Lima Perú; ^3^ Fred Hutchinson Cancer Research Center Seattle WA USA; ^4^ Epicentro Lima Perú; ^5^ Asociación Vía Libre Lima Perú; ^6^ Unison Medicare and Research Centre Mumbai India; ^7^ Harvard University Boston MA USA; ^8^ The Fenway Institute Boston MA USA

**Keywords:** drug resistance, Latin America and the Caribbean, transgender people, men who have sex with men, Peru

## Abstract

**Introduction:**

Transmitted, or any pretreatment drug resistance (TDR, PDR) can compromise efficacy of first‐line antiretroviral therapy (ART). In Peru, genotypic resistance testing is not routinely performed before ART initiation, and estimated PDR prevalence prior to 2012 ranged from 1.0% to 4.7%. We aimed to update estimates of PDR prevalence in men who have sex with men (cis‐MSM) and transgender women (TW).

**Methods:**

We obtained HIV sequences from three studies of ART‐naïve cisgender‐MSM and TW (n = 470) in Lima, Peru from 2013 to 2017, almost two‐thirds of whom had acute or recent infections. Sanger sequences of HIV *pol* were interrogated for surveillance drug resistance mutations (SDRM) using the Stanford Calibrated Population Resistance (CPR) tool and scored for resistance to nucleoside reverse transcriptase inhibitors (NRTIs) and non‐nucleoside reverse transcriptase inhibitors (NNRTIs) with the HIVdb programme. We calculated binomial proportions and 95% confidence intervals. χ^2^ and exact or trend tests were used to examine predictors of PDR.

**Results:**

Seventy‐seven (16.4%) individuals had PDR (95% CI: 13.2 to 20.0); most resistance was likely TDR since 63% were incident infections. SDRM were present in 9.8% (7.3 to 12.9). Resistance to any NRTI was present in <1% of individuals, while efavirenz resistance was present in 10% (6.9% to 12.4%). TW were not statistically more likely than cis‐MSM to have PDR (11.4% vs. 9.1%, *p* = 0.54). Age, incident versus prevalent infection, or residence district did not predict PDR. Prevalence of SDRM increased from 3% in 2013 to 21% 2017 within incident infections (*p* = 0.04), but not when including prevalent infections.

**Conclusions:**

Prevalence of NNRTI resistance in three studies of ART‐naïve MSM and TW in Lima, Peru reaches 10%. Because our study reports PDR in a population in which most acquired HIV recently, the overall prevalence of PDR, including previously treated persons, is likely underestimated. These results underscore the need for a nationally representative survey of PDR in Peru and consideration of non‐NNRTI anchored first‐line ART options. This study also represents the first evaluation of PDR in cis‐MSM versus TW in South America, and demonstrates that, although TW are at higher risk of acquiring HIV, they are at similar risk of acquiring a virus with resistance mutations.

## 1 Introduction

The ability to successfully treat HIV‐1 infection is contingent upon the continued efficacy of antiretroviral drugs (ARVs). Of 36.9 million persons living with HIV (PLWH) worldwide, 21.7 million (or about 60%) were accessing combination antiretroviral therapy (ART) as of 2017 1. Acquisition of much HIV drug resistance (HIVDR) is attributed to poor ART adherence, leading to periods of sub‐therapeutic ARV levels and functional mono‐ or dual‐therapy, which can ultimately result in virologic failure (acquired drug resistance (ADR)) 2, 3. Other causes of ADR include use of the non‐fully suppressive regimens available prior to the mid‐1990s, intermittent provision of ARVs, including prior peripartum prevention approaches 4, 5, 6, 7, or problems with consistent ART supply chain 8, 9. Transmitted drug resistance (TDR) refers to HIVDR present when a new HIV infection is acquired, whether through horizontal or vertical transmission; pretreatment drug resistance (PDR) is the broader term encompassing TDR and any HIVDR present at the initiation or re‐initiation of ART, and is more commonly used to describe infections of unknown duration and when TDR cannot be conclusively ascertained. PDR can result in failure of first‐line regimens, especially when not recognized at time of ART initiation 10. The WHO currently recommends initiation of ART regimens not anchored on non‐nucleoside reverse transcriptase inhibitors (NNRTI) when PDR exceeds 10%, or else genotypic testing for HIVDR prior to ART initiation if not feasible to empirically use non‐NNRTI regimen 11. Testing for PDR is currently the standard of care in high‐income countries including the US 12, 13.

Peru instituted a National ART Programme in 2004 after the introduction of ART in the country in 1999 14. This programme has provided free access to ART for PLWH since 2006. At present, first‐line ART regimens in Peru include a combination of two nucleoside reverse transcriptase inhibitors (NRTI) with one non‐nucleoside reverse transcriptase inhibitor (NNRTI), generally efavirenz (EFV); the estimated ART coverage in Peru in 2018 was 73%, compared with 31% in 2010 15. These regimens are initiated empirically without routine genotypic testing for PDR. Previously reported prevalence in Peru ranges between 1% TDR from a study of a mixed population in Lima with data acquired before 2009 (n = 96) 16 to a high of 4.7% PDR from a study of men who have sex with men (MSM) from several Andean countries, including Peru, in 2009 (n = 149) 17. The Peruvian Sentinel Surveillance Survey from 2002 to 2003 reported a prevalence of 3.3% PDR in MSM from six cities (n = 359) 18. In its most recent report of HIV drug resistance, the WHO reported PDR in nationally‐representative ART naïve populations in Argentina, Brazil, and Colombia to be between 9.8% and 12.8%, with estimates increasing over time 19, although data from Peru have never been included in these reports due to the limited availability of surveillance data.

In Peru, cisgender MSM (cis‐MSM) and transgender women (TW) comprise the majority of PLWH. The HIV prevalence within TW has been estimated to be as high as 30% 20, 21, while the overall HIV prevalence in the Peruvian population aged 15 to 49 is 0.3% 22. Additionally, the HIV epidemic is concentrated in the capital, where not only the highest concentration of cis‐MSM live, but 22% of cis‐MSM are living with HIV; national HIV prevalence in cis‐MSM is approximately 12% 22, 23. Although cis‐MSM and TW represent the majority of PLWH in Peru, these groups have frequently been grouped together in studies of high‐risk populations, even though they largely have separate sexual networks. Because it is uncommon for TW to partner with cis‐MSM or other TW, and their partners may be less likely to be engaged in HIV care 24, it is possible that there is a differential risk of TDR between cis‐MSM and TW. Demonstration of increasing PDR within Latin American countries emphasizes the need for broader surveillance to inform policy and guidelines, inclusion of data from Peru, and separate reporting of data for unique risk populations 16.

## 2 Methods

We obtained HIV‐1 sequences and corresponding participant data from three completed parent studies in Lima, Peru: *¿Sabes?: HIV Testing and Treatment to Prevent Onward HIV Transmission among MSM and Transgender Women in Lima, Peru* (“*Sabes*”) 25, Spatial and Phylogenetic Clusters of HIV Microepidemics Among MSM in Lima (“Microepidemics”), and Gender‐Affirmative Transgender Care to Improve the HIV Treatment Cascade (“Feminas”) 26. *Sabes* is a multi‐step longitudinal study using a Seek‐Test‐Treat‐Retain (STTR) strategy to reduce community viral load to decrease HIV transmission (2013 to 2016, n = 3337). Participants in this study who screened HIV‐negative at Step 1 and consented to Step 2 (n = 2109) underwent monthly antibody/antigen testing and HIV RNA testing if seronegative, such that the timing of most infections was precisely known. Participants with acute or recent (within three months) infection detected in Steps 1 and 2 were eligible to join the Step 3 ART interventional study. We obtained sequence data from all Step 3 eligible participants (n = 256) as well as participants with incident infection of longer or unknown duration (n = 111). *Microepidemics* employed mobile testing vans dispatched to nightclubs and plazas in 2016 to 2017 to increase testing of high‐risk MSM and characterize HIV transmission “hot‐spots” by combining viral phylogenetics and geospatial mapping of neighborhoods and social venues in Lima. New HIV cases were defined as those for whom no prior positive data relating to HIV care, including ART use, were documented in the Peruvian national laboratory database. In this analysis, only participants with new HIV diagnosis and no prior record in the national ART programme were included to exclude possibility of prior ARV exposure. Lastly, *Feminas* was a study of gender affirming medical services coupled with HIV testing and ART for TW in Lima (2016 to 2017); transgender women who were naïve to ART and desired feminine hormone therapy were enrolled to assess whether co‐provision of these services could improve engagement in care. As part of each parent study, HIV‐1 sequencing was performed on cryopreserved plasma from the first phlebotomy at HIV diagnosis. We included in this analysis all amplifiable sequences from persons in these three studies who had either been newly diagnosed during the study or who had a recent diagnosis of HIV, determined to be ART‐naïve and viremic.

This study was considered not human subjects research by the University of Washington Human Subjects Division, as all data was previously collected under the parent studies and deidentified prior to transfer to our institution. All parent studies received appropriate ethical approvals by the local and/or national Peruvian and US‐partner institutions.

### 2.1 Laboratory methods

The cDNA HIV *pol* sequences were reverse transcribed from viral RNA extracted from previously‐unthawed cryopreserved plasma, with Sanger sequencing as described previously 27. Amplification primers IBR2_M and MozFO_M were used to extend the 2510 to 3209 region of the HIV *pol* gene. Sequencing included the region of reverse transcriptase (RT) relevant for most clinically relevant drug‐resistance mutations (DRM) to NRTIs and NNTRIs; K238T/N, Y318F, and N348I mutations therefore were unlikely to be captured. Because sequence data were originally obtained for phylogenetic analysis, regions coding protease and integrase were not sequenced.

Prevalence of resistance mutations was assigned by the Calibrated Population Resistance Tool (CPR) 28, based on the WHO surveillance drug resistance mutation (SDRM) list 29. Per WHO recommendations, we then used the Genotypic Resistance Interpretation Algorithm – HIVdb Programme (HIVdb, Stanford University, Stanford, CA) to calculate penalty scores for relevant NRTI and NNRTI, and sequences were determined to be either susceptible (<15, including potential low‐level resistance) or resistant (≥15; low‐, medium‐, or high‐resistance) as well as to report all identified DRM (including other polymorphisms) 30. Sequences resulting in a mixed call at a given codon were given the highest score for a corresponding mutation. Consensus sequences from all genotyped individuals were aligned and manually edited, and neighbour‐joining phylogenetic trees were used to seek evidence of laboratory contamination.

### 2.2 Statistical analysis

We defined incident HIV infections as those in participants who were either seronegative at diagnosis (RNA or p24+ only; acute HIV) or else seropositive with confirmed negative test within the preceding six months. Any infection of known duration >6 months or seropositive without prior testing history was considered a prevalent infection. Kruskal‐Wallis and χ^2^ tests were used for descriptive statistics. SDRM and total PDR prevalence was determined using binomial proportions and 95% confidence intervals. χ^2^, trend, and Fisher's exact tests examined relationships between SDRM and gender identity, year of sample acquisition, diagnosis as incident or prevalent infection, age category, and residence district. For analysis of trend by year of sampling, we excluded persons whose first positive test was prior to 2013 (n = 5). We categorized residence district, as reported by *Sabes* and *Feminas* participants, into five geographic regions of the metropolitan area (Lima Norte, Lima Centro, Lima Sur, Lima Este, and Callao), containing all 43 administrative districts. We performed a Fisher's exact test to examine if there was heterogeneity between resistance patterns acquired by TW versus cis‐MSM populations. All statistical analysis was completed in the R package 3.5.0 using R‐Studio 1.1.453 31, 32.

## 3 Results

We obtained 471 successfully‐amplified sequences from plasma. We excluded 1 sample from an individual who reported first diagnosis of HIV infection in 1995. Samples were obtained as part of HIV testing at medical clinics or HIV testing sites (86%) or different LGBTQ social venues or plazas (14%). Two hundred and ninety‐nine (64%) sequences were obtained from participants with acute or incident infections (≤180 days since last negative test), and 171 (36%) were from prevalent infections (>180 days or unknown duration). Characteristics of participants, by parent study, are found in Table 1. Overall, 140 (30%) participants were TW and 330 (70%) were cis‐MSM. HIV‐1 infection was predominately with subtype B (n = 452). Additional subtypes and circulating recombinant forms (CRF) included A (n = 3), BC (n = 1), BF (n = 6), C (n = 1), CRF12_BF (n = 1), CRF44_BF (n = 5) and F (n = 1).

**Table 1:** Characteristics of participants from the three parent studies

	Study			*p*‐value
	Sabes	Microepidemics	Feminas	
Total participants (%)	367	64	39	
Gender Identity				
Cisgender male	284 (77)	46 (72)	0 (0)	<0.0001
Transgender female	83 (23)	18 (28)	39 (100)	
Age				
18 to 25	195 (53)	34 (53)	20 (51)	<0.0001
26 to 35	138 (38)	22 (34)	15 (38)	
36 to 45	25 (7)	6 (9)	3 (8)	
46+	9 (2)	2 (3)	1 (3)	
Duration of Infection				
Incident (≤180 days)	282 (77)	6 (9)	11 (28)	<0.0001
Prevalent (>180 days)	85 (23)	58 (91)	28 (72)	
Year of sample				
2013	61 (17)	0 (0)	0 (0)	<0.0001
2014	133 (36)	0 (0)	0 (0)	
2015	134 (37)	0 (0)	0 (0)	
2016	39 (11)	0 (0)	22 (56)	
2017	0 (0)	64 (100)	17 (44)	
Initial viral load (log_10_ copies/mL)				
Median (IQR)	5.9 (5.1 to 6.7)	5.2 (5.0 to 5.8)	4.9 (4.6 to 5.1)	0.37
Initial CD4 + Count (cells/mL)				
Median (IQR)	416 (278 to 567)	347 (299 to 398)	323 (260 to 562)	0.63

Percent totals may not equal 100 due to rounding.

### 3.1 Pre‐treatment drug resistance

Looking first at all DRM (via HIVdb programme), a total of 92 DRM were identified across all sequences; this represented 22 unique base changes, 14 of which confer potential resistance to NNRTIs and 8 to NRTIs. Seventy‐seven individuals (16.4%) had at least one DRM (95% CI: 13.2 to 20.0) (Table 2). SDRM were present in 46 individuals (9.8%; 7.3 to 12.9). The most prevalent SDRM were K103N/S, G190A/E and M184V. Five thymidine analogue mutations (TAMs) were identified within two individuals, including Type II (n = 4), and Type I (n = 1) TAM pathways. Seven (1.5%) individuals had 2 or more unique mutations and Genotype Susceptibility Score (GSS) to first‐line regimens was ≥2 except in three individuals overall. There was no difference in prevalence of TDR between the three parent studies (*p* = 0.30). 

**Table 2:** Prevalence of Drug Resistance Mutations among 470 ART‐naive participants

Type	# With mutations	Prevalence (95% CI) (n = 470)	
Total with any HIVDR	77		16.4% (13.2 to 20.0)
Total with SDRM	46		9.8% (7.3 to 12.9)
NNRTI SDRMs			9.3% (6.9 to 12.4)
K103N/S	34		7.4% (5.2 to 10.2)
G190A/E	6		1.3% (0.5 to 2.8)
K101E	3		0.6% (0.1 to 1.9)
Y181C	1		0.2% (0.0 to 1.2)
Y188C	1		0.2% (0.0 to 1.2)
NRTI SDRMs			1.3% (0.5 to 2.8)
M184V	4		0.9% (0.2 to 2.2)
T215F/Y	2		0.4% (0.1 to 1.5)
D67N	1		0.2% (0.0 to 1.2)
K70R	1		0.2% (0.0 to 1.2)
L74I	1		0.2% (0.0 to 1.2)
V75M	1		0.2% (0.0 to 1.2)
K219Q	1		0.2% (0.0 to 1.2)
Any TAM	5		1.1% (0.3 to 2.5)

HIVDR, HIV Drug Resistance; SDRM, Surveillance Drug Resistance Mutations; TAMs, Thymidine analogue mutations, including both Type I and Type II.

Incorporating the cumulative effects of all DRM in a sequence, each participant was scored by HIVdb as either susceptible or resistant against 5 NRTIs currently used or considered for future use in Peru (abacavir (ABC), azidothymidine (AZT), emtricitabine (FTC), lamivudine (3TC), tenofovir disoproxil fumarate (TDF)) and 4 NNRTIs (efavirenz (EFV), nevirapine (NVP), rilpivirine (RPV) and doravirine (DOR); Table 3). Resistance to any NNRTI ranged from 5.0% (RPV) to 12% (NVP), while NRTI resistance was substantially lower, between 0.2% (TDF) and 0.9% (ABC, 3TC/FTC).

**Table 3:** Frequency of resistance to commonly used nucleoside and non‐nucleoside reverse transcriptase inhibitors (NRTI and NNRTI)

	Antiretroviral drugs								
	NRTI					NNRTI			
ARV	ABC	AZT	FTC	3TC	TDF	EFV	NVP	RPV	DOR
Susceptible	466	468	466	466	469	426	418	448	441
Resistant	4	2	4	4	1	44	52	22	29
Overall % Resistance (95% CI)	0.9 (0.2 to 2.2)	0.5 (0.1 to 1.5)	0.9 (0.2 to 2.2)	0.9 (0.2 to 2.1)	0.2 (0.0 to 1.2)	10.0 (6.9 to 12.4)	12.0 (8.4 to 14.3)	5.0 (2.0 to 7.01)	6.2 (4.2 to 8.7)

ABC, abacavir; AZT, azidothymidine; FTC, emtricitabine; 3TC, lamivudine; TDF, tenofovir disoproxil fumarate; EFV, efavirenz; NVP, nevirapine; RPV, rilpivirine; DOR, doravirine.

### 3.2 Predictors of SDRM

**Figure 1:**
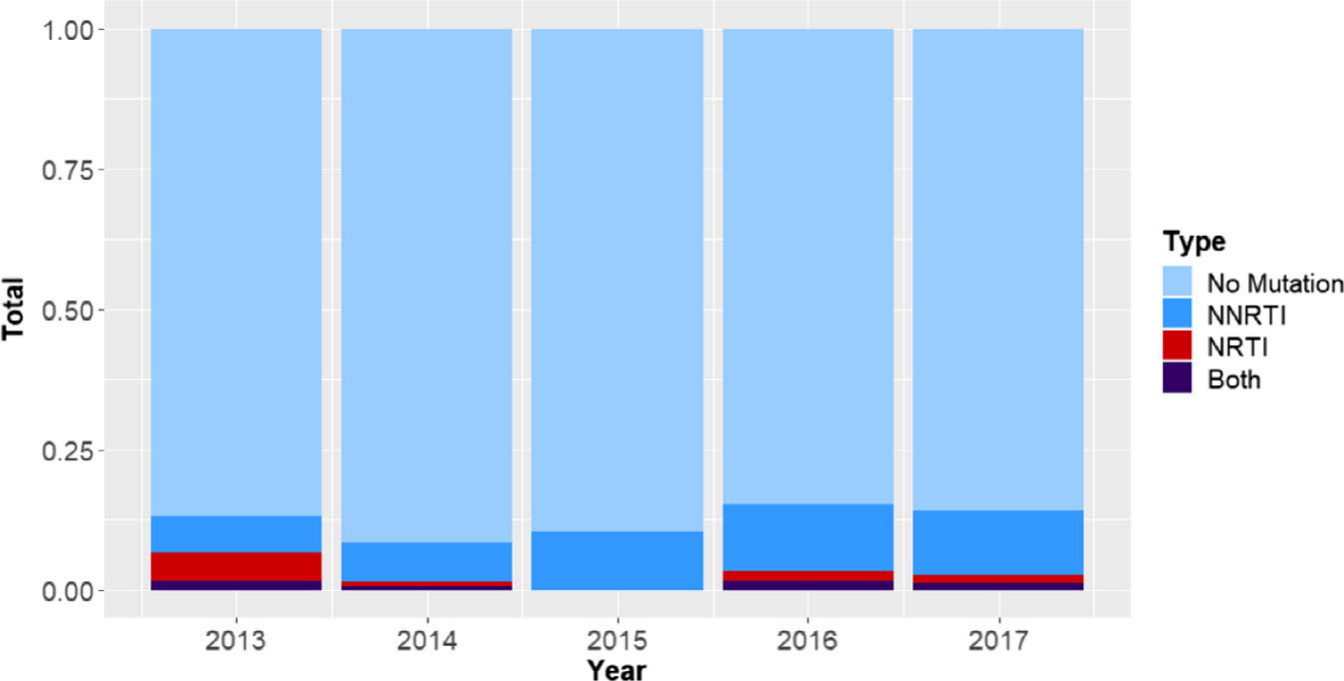
**Surveillance drug resistance mutation prevalence by year**. Percent and type of mutation for each sample year excluding five individuals with known positive date preceding study years.

There was no difference in the likelihood of SDRM between incident and prevalent infections (9.4% vs. 10.5%, *p* = 0.75). There was 91.3% concordance between year of HIV diagnosis and year of sample acquisition. After excluding five samples from participants who reported HIV diagnosis prior to 2013, there was no significant trend in overall SDRM prevalence between 2013 and 2017 (*p* = 0.33 for trend, Figure 1). Restricting this analysis to incident HIV diagnoses (n = 299), there was a significant increase across this period, (3% in 2013, 7% in 2014, 11% in 2015, 11% in 2016 and 21% in 2017; *p* = 0.04). The prevalence of SDRM in TW was 11.4% (95% CI: 6.7 to 17.9), which was not significantly higher than cis‐MSM at 9.1% (95% CI: 6.2 to 12.7), *p* = 0.54. However, we detected heterogeneity in the pattern of DRM between TW and cis‐MSM, meaning that observed mutations were not evenly distributed between gender identity groups (*p* < 0.0001). The following mutations were more common in the cis‐MSM population V108I, E138A and V179D; V179E and G190A were more common in TW.

Analysis by age likewise suggested no difference in PDR by age group (*p* = 0.82 for trend across age groups). Four‐hundred and five participants (86%) had residence district information available. Whether evaluated by individual residence district or the larger five regions of Lima, we also found no heterogeneity in risk of PDR (*p* = 0.57), including no single district with a statistically elevated risk of PDR.

## 4 Discussion

This study leveraged HIV sequence data from three studies of cis‐MSM and TW to complete the largest and most recent evaluation of PDR to our knowledge in Peru. Compared to prior studies in Peru estimating TDR or PDR at 4% or below, we observed 9.8% prevalence of SDRM and 16.4% prevalence of all DRM – nearly threefold higher than the previously documented results in 2009 17. The prevalence of resistance to the two currently available NNRTIs, EFV and NVP, were 10% and 12% respectively. Additionally, while prior estimates from Peru were lower than those from neighbouring countries, our observed PDR approximates the most recent nationally representative WHO PDR estimates for counties such as Argentina 12.8% (9.2 to 17.4), Brazil 9.8% (8.1 to 12.0) and Colombia 9.9% (7.5 to 12.9), despite the fact that our study only includes ART‐naïve persons, does not include PI mutations, and excludes cisgender women, who have the highest rate of PDR globally 19. In addition to increased PDR prevalence in our study compared to older data, the prevalence also increased across study years within the present report.

As in many other low‐ and middle‐income countries that adopted broad use of ART only after highly active combinations were available in the mid‐2000s, the most common DRMs detected confer resistance to NNRTIs, rather than NRTI or other classes. Higher prevalence of NNRTI compared to NRTI resistance is expected due to factors such as the pharmacokinetics of these drug classes, differential impact of DRM on viral fitness and therefore persistence of these strains, and less significantly in Peru, the prior use of single‐dose nevirapine in prevention of mother‐to‐child transmission of HIV (PMTCT). Because the epidemic in Peru is largely in persons assigned male sex at birth, and HIV prevalence in cisgender women is 0.2% overall 21, the high rate of NNRTI resistance is likely more attributable to adherence and supply‐chain consistency 8, 9. Because at the time of this study, HIV pre‐exposure prophylaxis (PrEP) with oral TDF/FTC was only available through studies and demonstration projects in Lima, our data is unlikely to show influence of PrEP on TDR, but is reassuring that TDF/FTC should retain high efficacy given the <1% observed prevalence of NRTI resistance.

We acknowledge certain limitations of this study. Our data examine HIVDR related to NRTI and NNRTI, but we were unable to investigate mutations that confer resistance to protease inhibitors (PI) or INSTI as available sequences lacked the gag/pol region coding for protease or integrase genes. At present however, neither PIs nor INSTIs are included in first‐line ART for adults in Peru, and so PDR to these classes is unlikely. Additionally, these data are based on high‐risk populations in Lima most of whom had incident HIV and were confirmed to be ART‐naive, and do not comprise a systematic national‐level PDR survey, which should include cisgender women and persons re‐initiating ART. However, with 90% of the HIV epidemic believed to be encompassed within MSM and TW communities, and largely within Lima, this sample is likely representative of TDR in PLWH at present in Peru 22, 23, 33. Additionally, two of three prior studies on PDR in Peru were focused in these same populations; thus, our estimates are comparable to these published data. Because we limited our analysis to newly diagnosed individuals most of whom had recently acquired HIV, we likely underestimate PDR, which can include previously treated individuals. Inclusion of such individuals would likely increase estimates to levels at or above other Latin American countries. Although not unique to our study, social desirability bias can cause participants to omit report of previous ART receipt. We mitigated this bias by cross‐referencing prevalent infections with the national ART programme database.

This study has several strengths, including the large sample size drawn from three different source studies, including participants recruited from studies offering HIV testing in both the clinic setting and at social venues, including dance clubs, saunas, plazas, and a programme featuring outreach specifically to TW. Therefore, we were able to access hidden populations that may not be included in testing of routine samples from ART‐initiation visits, which biases towards groups more likely to link to care. Additionally, the large proportion of incident infections and careful attention to verifying prior HIV testing history allowed us to largely exclude persons with long‐standing infections or prior ART exposure. The high concordance between year of sample with probable timing of infection allowed us to evaluate trends in new infections over time. We also performed all sequencing in an expert reference lab, which is currently otherwise unavailable in Peru. Lastly, we present the first differentiated estimates for TW and cis‐MSM populations in South America, which is important given the low overlap between these two populations. Since TW mainly partner with heterosexual‐identifying cisgender men 24, TDR in this population may better reflect infections that bridge to non‐MSM populations.

## 5 Conclusions

These data highlight the importance of ongoing monitoring of HIVDR as suggested by the WHO 34. The high prevalence of NNRTI mutations suggests that adoption of INSTI‐based regimens for first‐line therapy could be prudent, especially for cis‐MSM and TW. In the case of dolutegravir, the concern about possible neural tube defects would not be relevant in these populations 35. The risk of NRTI mutations remains low, even in comparison to Brazil 36, which recently reported a similar estimate for overall TDR but a threefold higher proportional risk for NRTI DRM. Our data therefore show little threat to the efficacy of NRTIs in current use in ART or as PrEP. Overall, the prevalence of TDR in cis‐MSM and TW in Lima is much higher that previously recognized, and despite different socioeconomic status, HIV incidence, and access of health and HIV services between these two populations, we found little difference in the risk of transmitted drug resistance in recent infections. Further geographically representative data from cisgender women, children and heterosexual men should be collected to inform national and regional ART programmes.

## Competing interests

No authors report competing interests.

## Authors' contributions

WLT performed all statistical analysis and wrote the initial manuscript. JRL, AD, HS, RC, TG, SLR and KHM designed and executed the parent studies, provided data and gave input to results and implications. PS provided input from the national ART programme. JM performed all laboratory work for the parent studies and assisted in interpretation of sequence data. RBI designed and assisted in the analysis and wrote the manuscript. All authors have read and contributed to the final manuscript.
